# Simulated optical performance of soft contact lenses on the eye

**DOI:** 10.1371/journal.pone.0216484

**Published:** 2019-05-14

**Authors:** Ahmed Abass, Samantha Stuart, Bernardo T. Lopes, Dong Zhou, Brendan Geraghty, Richard Wu, Steve Jones, Ilse Flux, Reinier Stortelder, Arnoud Snepvangers, Renato Leca, Ahmed Elsheikh

**Affiliations:** 1 University of Liverpool, Liverpool, United Kingdom; 2 University of Toronto, Toronto, Canada; 3 The Federal University of São Paulo, São Paulo, Brazil; 4 Central Taiwan University of Science and Technology, Taichung, Taiwan; 5 Pacific University, College of Optometry, Forest Grove, Oregon, United Sates of America; 6 Eaglet Eye B.V., Houten, The Netherlands; 7 Faculdade de Medicina do ABC (FMABC), São Paulo, Brazil; 8 National Institute for Health Research (NIHR) Biomedical Research Centre at Moorfields Eye Hospital NHS Foundation Trust and UCL Institute of Ophthalmology, London, United Kingdom; 9 School of Biological Science and Biomedical Engineering, Beihang University, Beijing, China; Cardiff University, UNITED KINGDOM

## Abstract

**Purpose:**

To evaluate the impact of soft contact lens eye-fit on optical power by computational modelling and to produce correction maps for reversing this impact during the design process.

**Methods:**

Finite element models of spherical and toric hydrogel contact lenses at varying nominal powers of -20 D to +20 D, base curves radii (R_1b_) of 8.2, 8.5, 8.8 mm, and overall diameters (d_3_) of 14.5, 15.0, 15.5 mm were generated. Lenses were fitted to computational eye models generated with human eyes’ topography data. Combined eye-lens simulations were run under the boundary conditions of the tears’ surface tension between the contact lens and the eye in addition to the eyelid blink pressure. Lens optical zone power changes were calculated through computational light-ray tracing methods following each simulation.

**Results:**

Effective power changes (EPC) were affected negatively for all toric simulated lenses with power varying from -20 D to +20 D. Spherical lenses demonstrated similar behaviour, however with some positive EPC over the power range from -20 D to -10 D for spherical power (SPH) lenses. EPC assessment was between +0.25 D and -0.5 D for most lenses, however, lenses with prescriptions from +10 D to +20 D incurred EPC outside this range. The spherical lenses showed a maximum effective power change of +1.046 ± 0.338 D (Average Eye), and a minimum of -3.278 ± 0.731 D (Steep Eye). Similarly, the toric lenses showed a maximum of +1.501 ± 0.338 D (Average Eye), and a minimum of -3.514 ± 0.731 D (Steep Eye). EPC trends, along with minimum and maximum power, generally increased negatively as nominal lens prescription increased positively. Contact lens base curve selection affected the assessed effective power change for both spherical and toric lenses. The effect from lens total diameter for spherical lenses was less substantial than that for toric lenses.

**Conclusions:**

This study considered the impact of soft contact lens design parameters on effective optical power changes (EPC) after eye-fit. Spherical lenses experienced more EPC of clinical significance (>0.25 D) than toric lenses. Both types of lenses, spherical and toric (simple astigmatism), demonstrated similar trends in EPC on fitting from -20 D to +20 D, with lenses in the extremely positive and the extremely negative prescriptions demonstrating the highest EPCs. The lens base curve impacted the extent of EPC observed, with flatter base curves experiencing less power change. Diameter proved to impact toric lenses more than spherical ones, however generally the diameter has less effect on power change than base curve selection.

## Introduction

When a contact lens is placed on the eye, the effects of the eyelid interaction and the tears’ surface tension change the lens dimensions and therefore alter its predesigned refractive power. Changes in the optical power of soft contact lenses during the fitting process have been reported by researchers for more than four decades, however, it is not yet possible to precisely predict the performance of clinical soft lens fitting [1–6]. Power changes occur as the surface of a lens undergoes relative changes in shape and thickness due to conformance with the cornea [1]. With this conformance the optical path through the lens is changed, consequently changing the focal length and power throughout the lens optic zone. The degree to which the optical power changes during fitting is known as the “supplemental power” [2] and is attributed to a combination of effects from shape change and tear film thickness. Strachan [7] treated the soft contact lens wrap-factor as a constant that can be calculated as the ratio between the lens base curve and the radius of the cornea [8]. With the absence of modern simulation analysis, Janoff [9] listed four theories that attempted to account for lens flexure on the refractive power of soft contact lenses and concluded a surprising finding that the soft lens flexure is similar to the bending of a metal beam. Knowing the fact that the average Poisson's ratio of metals is 0.3 and of the hydrogel is 0.49, it is very difficult to accept Janoff and Dabezies’s conclusion in an engineering sense.

Kollbaum [9] has suggested that further investigation of supplemental power change may be essential to achieve predictable and optimised lens design performance. This corroborates well with the recent work of Sulley et al. demonstrating the wide variation in theoretical fitting success rates of soft contact lenses (61% - 90%) despite a narrow range of parameters varying between commercial designs (Base curve radius 8.4 mm to 9.0 mm, and D from 13.8 mm to 14.3 mm) [10]. Historically, changes to optical power from corneal conformance have been estimated roughly. However, modern advances in computational modelling and finite element analysis techniques make these methods a better choice to study a complex eye-contact lens system.

In this study, computational models of varying hydrogel semi-scleral contact lenses and the human eye were generated via a MATLAB code. The non-linear finite element software FEBio was then used to simulate the models to monitor the lenses drape, stretch and settle onto the cornea. Lenses were subject to negative surface tension pressure in addition to the eyelid blink pressure in order to observe the changes to their optical power after being fitted to the eyes.

## Materials and methods

The presented study was approved by the ethics committee of the Federal University of São Paulo (Brazil) and was conducted in accordance with the standards set out in the Declaration of Helsinki. The participants’ data were collected from a previously built and anonymised database from Brighten Optix Corporation in Taipei, Taiwan where written consents for using their data for research purposes were signed before scanning their eyes. For this study, three eyes were selected from healthy participants’ record based on their geometry. Participants’ anterior eye profile data were only used to build finite element models of their eyes.

The optimal soft contact lens adaptation, which allows patients’ comfort, good quality of vision and minimal interference with ocular surface functions and metabolism, is the result of a delicate balance between eye and lens dimensions and mechanical properties. In order to simulate a precise contact lens fitting scenario, three sets of eye-lens systems were modelled in this study considering flat, average and steep corneas [11].

Participants’ anterior eye topography data was obtained via corneoscleral height Fourier profilometry captured by an Eye Surface Profiler (Eaglet Eye B.V., Houten, The Netherlands) and incorporated into patient-specific finite element models by a custom-built MATLAB software (MathWorks, Natick, USA) before being simulated in the FEBio nonlinear finite element analysis software (University of Utah, Salt Lake City, USA). The selection of suitable eyes for inclusion in the study was carried out in light of Gilani’s population study of eyes’ topography [12]. The median of the flat power simulated keratometry (Sim-K) was 43.8 D, and the bounds of “flat” and “steep” corneas were determined by applying one standard deviation of ±1.38 D [12]. By applying this classification to the patient data set, corneas were classified as “flat” if their flat meridian power was less than or equal to 42.4 D, “steep” for flat meridian power of 45.2 D or above, and average if it was in-between, Table 1. Full three-dimensional eyes’ surfaces are presented in S1 as X, Y and Z Cartesian coordinates.

**Table 1:** Selected eyes to model from participants’ data.

Subject ID	Eye classification	Eye side	Sex	Age	Flat Sim-K Power
Participant 1	Flat	Right	Female	28 year	41.8 D (8.07 mm)
Participant 2	Average	Left	Male	34 year	43.8 D (7.71 mm)
Participant 3	Steep	Right	Female	25 year	46.8 D (7.21 mm)

Soft semi-scleral contact lenses are typically fitted 0.3 mm to 1.0 mm flatter than the flattest corneal Sim-K [13]. Accordingly, “Flat” (R_1b_ = 8.8 mm), “Average” (R_1b_ = 8.5 mm) and “Steep” (R_1b_ = 8.2 mm) contact lens designs were paired with each eye. Base curves (R_1b_s) were selected based on commercially available OP42 soft contact lenses by Optolentes (Porto Alegre, Brazil). Clinically, soft semi-scleral lenses are typically fitted with overall diameters (d_3_) between 13.50 mm and 16.00 mm, with a step of 0.50 mm, to verify sufficient corneal coverage [13]. In a recent study of theoretical fitting characteristics by Sulley et. al. [10], it was identified that the biggest opportunity for achieving a soft contact lens fitting success was to increase the lens diameter, hence improving corneal coverage. Lenses tested in Sulley et. al.’s study represented 15 commercial soft contact lens brands with base curves (R_1b_) of 8.40 to 9.0 mm and overall diameter (d_3_) of 13.8 to 14.3 mm. Considering the importance of good corneal coverage, overall lens diameters (d_3_) were selected as 14.5 mm, 15.0 mm, and 15.5 mm for the current study.

The spherical and toric eye-lens systems simulated in this study are described in Table 2. As ‘with-the-rule’ astigmatism (which occurs when the corneal vertical meridian is steeper than the corneal horizontal meridian) is the most common shape of astigmatism, toric lenses in this study were designed with cylindrical power aligned on the vertical meridian, and zero power along the horizontal meridian, (see S2 & S3).

**Table 2:** Contact lenses design parameters.

Eye	Contact lens			
Classification	Base curve radius	Spherical power (SPH)	Cylindrical power (CYL) @ 90°	Diameter (d_3_)
Flat	8.8 mm	-20.0 D, -15.0 D, -10.0 D, -5.0 D, 0.0 D, 5.0 D, 10.0 D, 15.0 D, 20.0 D	-20 D, -15 D, -10 D, -5 D, -2.5 D, -1 D, 0 D, 1 D, 2.5 D, 5 D, 10 D, 15 D, 20 D	14.5 mm, 15.0 mm, 15.5 mm
Average	8.5 mm			
Steep	8.2 mm			

### Uniaxial tensile testing

**Fig 1:**
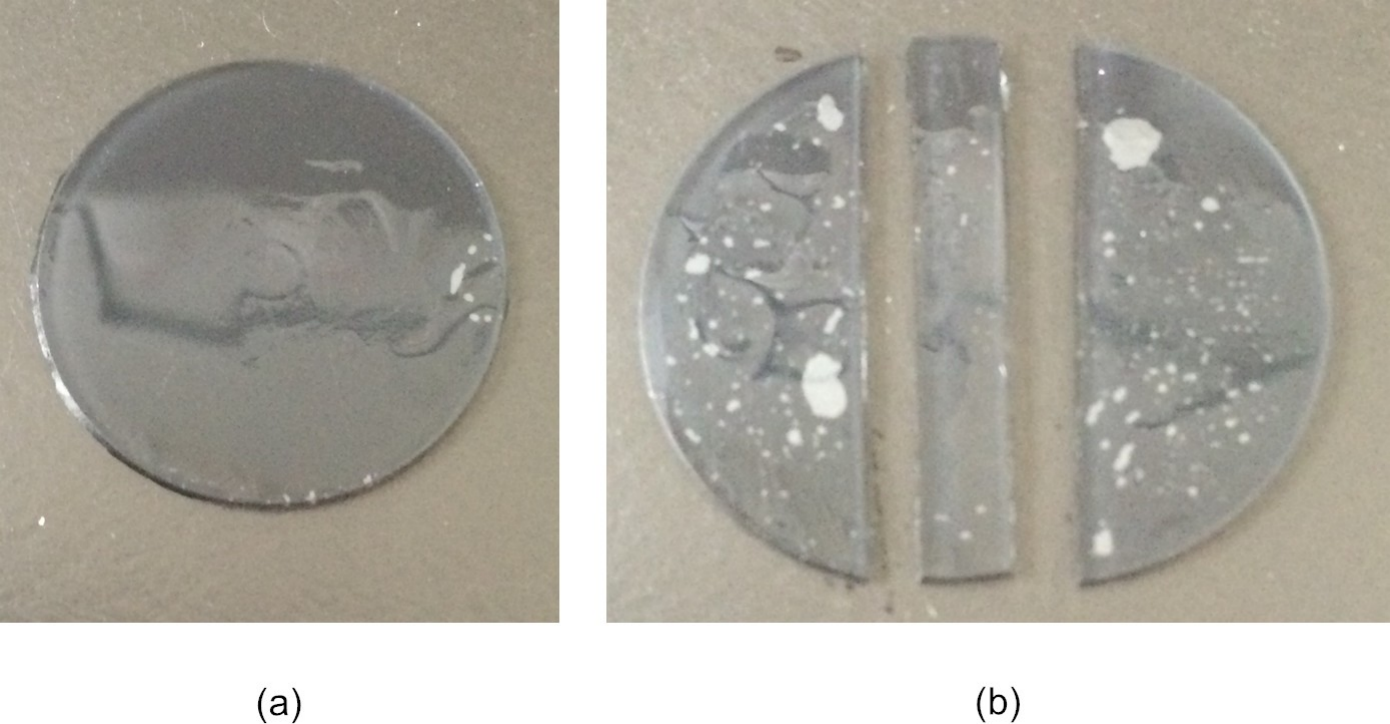
Hydrated clear hydrogel sample on a metallic silver thick paper layer underneath to ensure clear appearance in the photograph, (a) before the cut, (b) after the cut.

**Fig 2:**
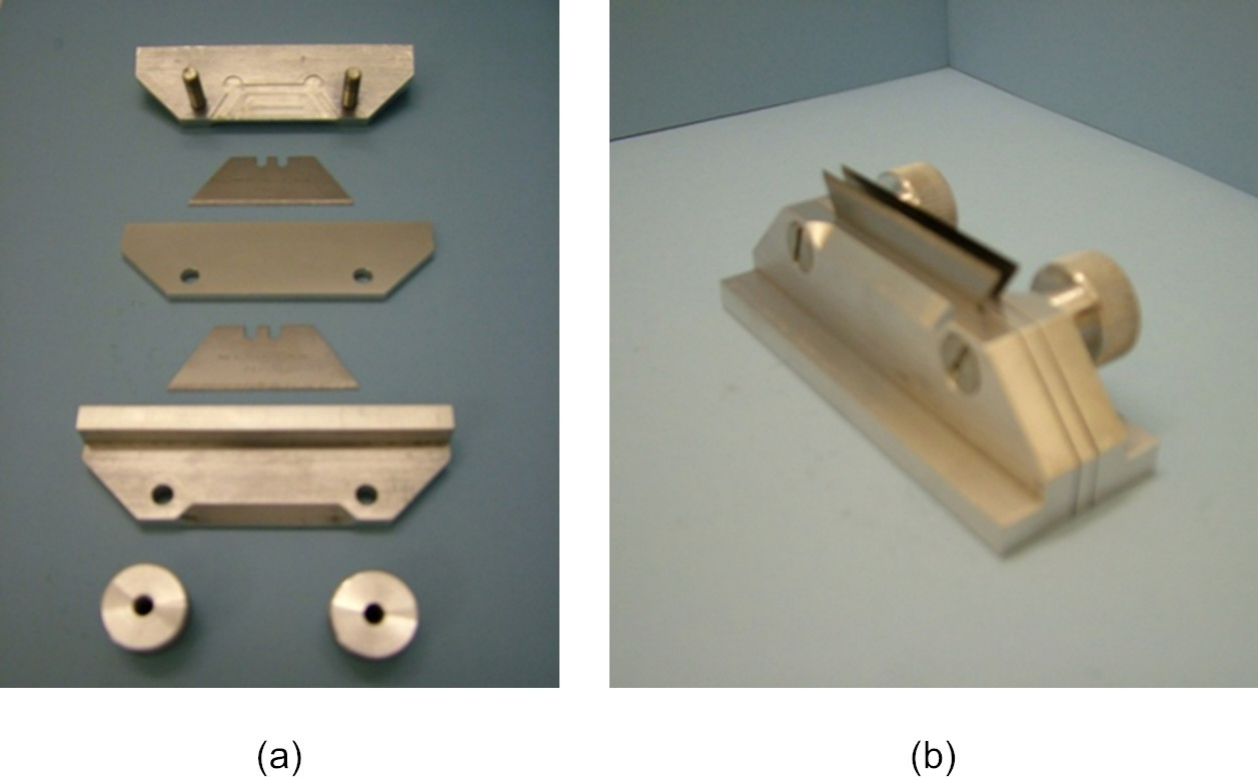
Double blade cutting tool used for strip extraction including (a) individual components and (b) assembled tool.

Three samples of non-ionic hydrogel material Filcon II3, with 77% water content (Contamac, Saffron Walden, England, UK) were tested experimentally at the Biomechanical Engineering lab at the University of Liverpool on an Instron 3366, dual-column, table-top materials testing machine equipped with a calibrated 10 N load cell. Uniaxial tensile tests were carried out using a modular, Bluehill3 software package, then processed by MS Excel software (Microsoft, Redmond, USA) and MATLAB software (MathWorks, Natick, USA) to determine the material’s properties. As raw contact lens material samples were in cylindrical shape (12 mm diameter & 6 mm thick) and the average water expansion of the hydrogel material was 1.61 in the radial direction and 1.635 in the axial direction, a centr lathe cutting machine was used to reduce the dry height to nearly 0.5 mm while the water-based coolant fluid temperature was kept at 20°C. Samples were hydrated for 8 hours in three glass containers filled with 0.90% Borate Buffered Saline (BBS) solution before being subject to a lightweight autoclaving process at 121°C for 20 minutes. After the hydration process, the three samples’ diameters were expanded to 20.7, 20.93 & 20.9 mm and thicknesses to 0.91, 0.88 & 0.8 mm respectively, Fig 1. Strips were cut using a custom-built adjustable-width double-bladed cutting tool that was designed to accommodate the desire for strips of varying width, Fig 2. Their lengths were measured using a digital Vernier calliper (D00352, Duratool, Taiwan) accurate to ±10 μm. Samples’ widths were measured as 3.34, 3.51 & 3.48 mm and their lengths were measured as 6.22, 6.15 & 5.89 mm respectively. Specimen width was measured at three equally-spaced locations along the test length and averaged. The thickness was measured using an electronic Vernier calliper (D00352, Duratool, Taiwan) at the same three locations and averaged.

**Fig 3:**
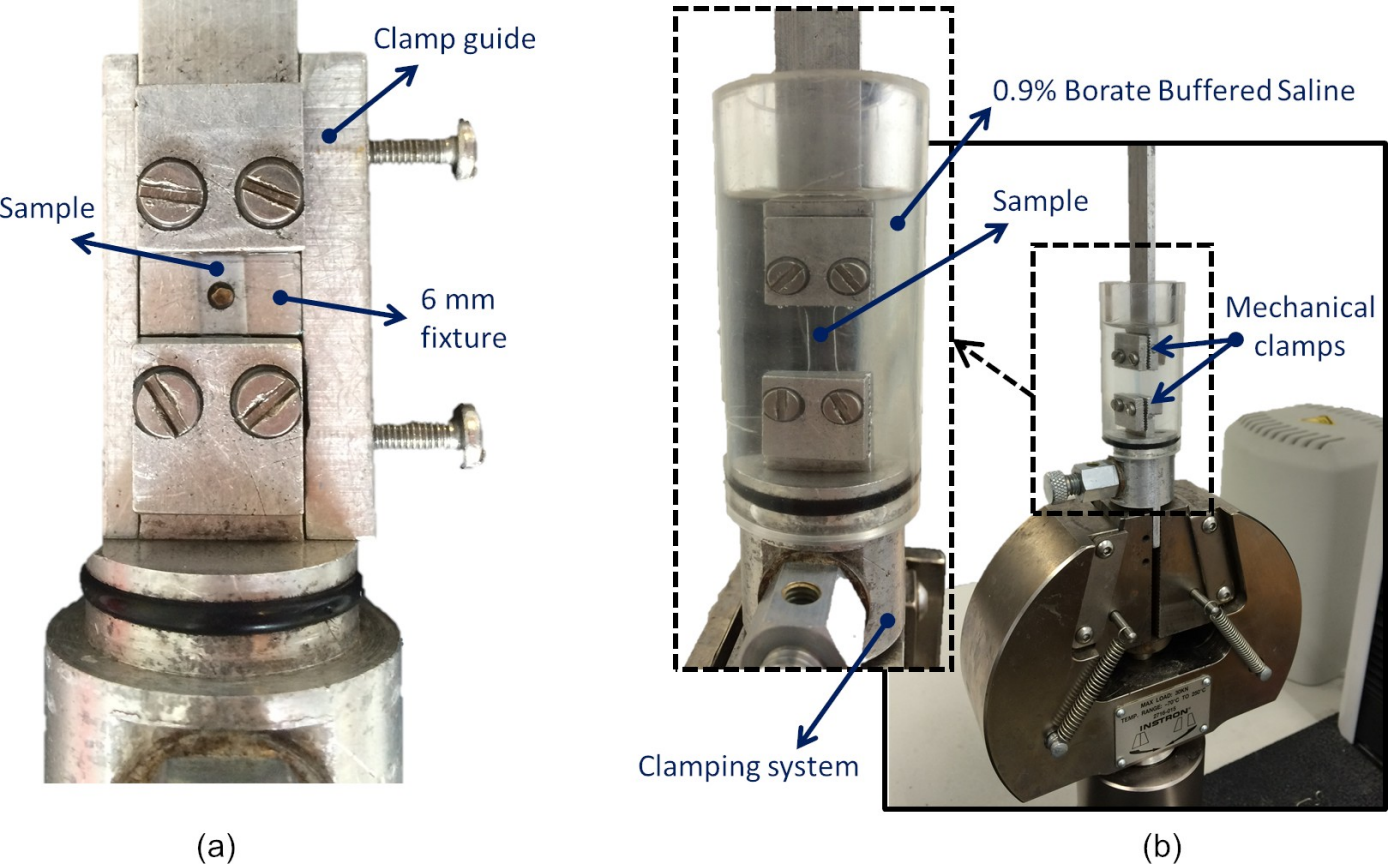
Test set up showing (a) sclera strip specimen attached to assembled clamps, (b) specimen fitted to mechanical clamps and connected to a material testing machine.

Specimens were subjected to ramp loading up to 1.0 N with a strain rate of 10% per minute. A Perspex tube chamber (Fig 3) was placed around the specimen and filled with purified water (ReAgent Chemical Services, UK) to maintain specimen hydration throughout the duration of the test. The mechanical tensile stress 𝜎_t_ was calculated by dividing the applied load *F* by the strip initial cross-sectional area *A* [14] as

$$\sigma_{t}=\frac{F}{A}$$ Eq 1

The strain *ε* was calculated as the ratio of change in the strip extension Δ*L*, which is the absolute difference between the initial strip length *L*_0_ and its instantaneous length, *L*, at the time of calculating the strain, over the initial length.

$$\varepsilon =\frac{\Delta L}{L_{0}}$$ Eq 2

**Fig 4:**
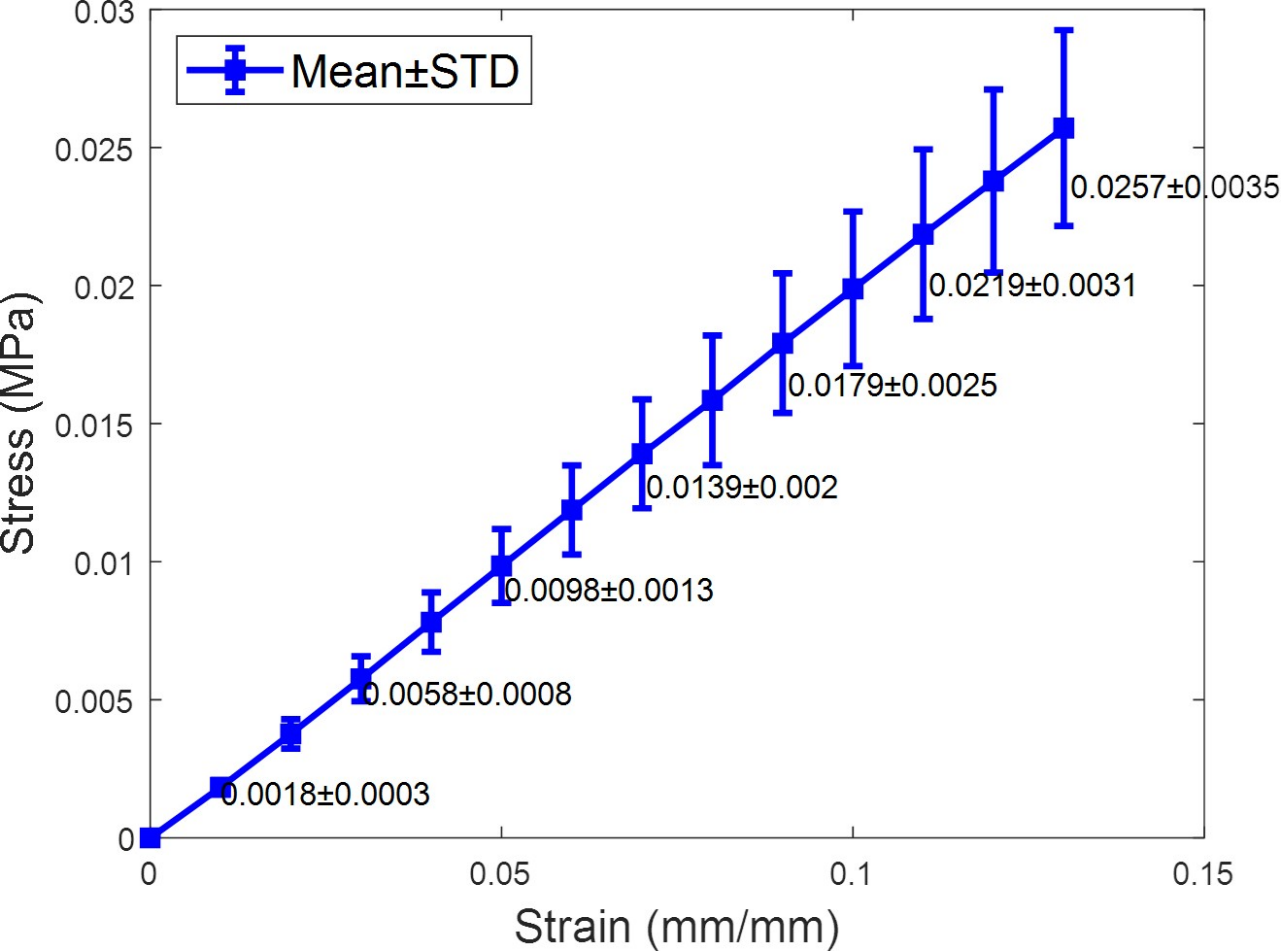
Mean stress-strain curve of 3 hydrogel samples.

As a linear behaviour was observed in the *ε*<0.15 deformation region, the elastic modulus of the material was determined as the slope of its stress–strain curve, Fig 4.

$$E=\frac{\Delta \sigma_{t}}{\Delta \varepsilon }$$ Eq 3

Subsequently, after testing three samples, the average elastic modulus of the material was determined as 0.199± 0.028 MPa, with a coefficient of variation (CV) well under one (0.141).

### Finite element modelling

Eye-lens systems were simulated with non-ionic hydrogel material Filcon II3, with 77% water content (Contamac, Saffron Walden, England, UK), a linear forced upper eyelid blink pressure of *P*_1_ = 8.0 mmHg was released linearly on the middle of the step time [15] and a surface tension of tear fluid of P_2_ = 43.6 mPa [16]. The finite element model was composed of two parts, the contact lens and the anterior eye, with a single interface between them. Contact lens was modelled as an incompressible linear elastic solid with young’s modulus 0.199 MPa and Poisson’s ratio 0.49. The anterior eye was assumed as rigid as the clinical investigations of the short-term effect of corneal soft contact lenses on the eye shape showed no effect [17]. As the used topography machine (Eaglet-Eye’s Eye surface profiler) which can cover the cornea and portion of the sclera is not capable of measuring the posterior surface of the eye, the back-surface of the eye model was formed as a parallel surface to the anterior eye, with a 545 μm constant thickness profile [18]. Eye anterior surface was taken as a master surface; however, the contact lens back-surface was taken as a slave surface with the gap tolerance set to zero. The anterior eye and contact lens were assumed as impenetrable bodies and the coefficient of friction was set to 0.01 consistently on the contact surface [19]. Modelling the tear film as an extra layer, as simulated by Day [20], was not considered in this simulation, however, the effect of the surface tension of the tears was modelled via the pressure *P*_2_. The eyelid blinking pressure *P*_1_ was imposed on the front-surface of the contact lens, however, tear’s surface tension pressure *P*_2_ was applied on the back-surface of it. However, the displacement of the eye was constrained in all directions, only the optical centre of the contact lens was constrained in the X and Y displacements.

**Fig 5:**
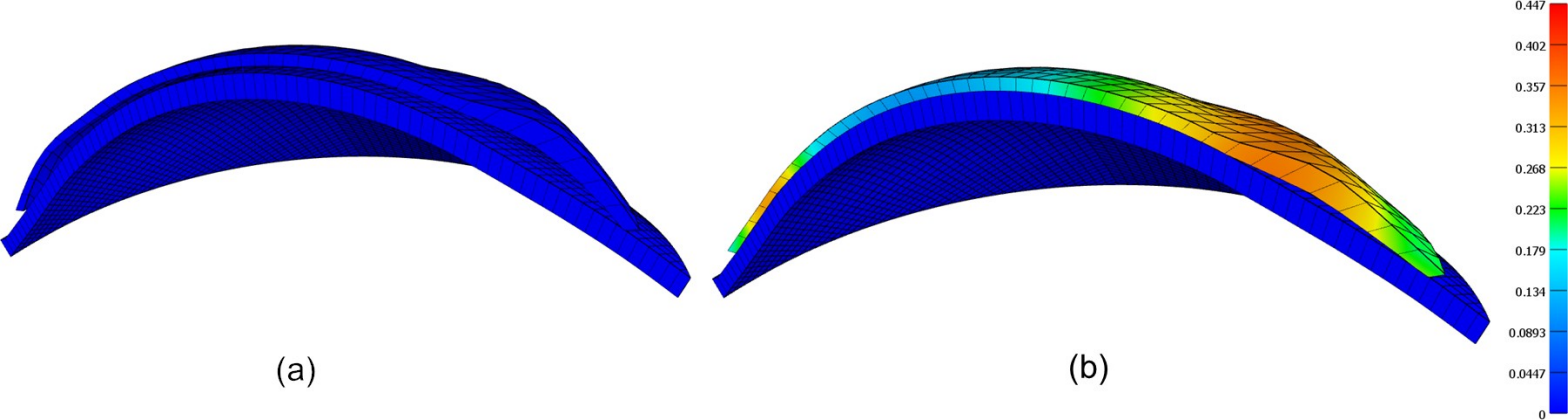
Contact lens finite element model, (a) before fitting, (b) after fitting where the maximum effective Lagrange strain at the final step of this simulation was 0.0768.

Both eye and contact lens were modelled using 3280 and 2278 eight-node trilinear hexahedral solid elements (HEX8), respectively, with 6882 nodes for the eye and 1842 nodes for the contact lens. The convergence study was carried out with 8 models of contact lenses with zero optical power. These models have several elements varying from 598 to 3808 per layer up to 2 layers only as more than 2 layers resulted in an inappropriate aspect ratio of elements’ dimensions and low mesh quality. The mesh convergence investigation showed -73% change in the displacement when the number of elements increased from 598 to 2278, while the much smaller change of displacement in -0.45% occurred up to 3808 elements. The outcomes showed that the number of the elements equal to 2278 arranged in one layer has converged to the displacement of 99.825 μm at the apex node and selected as an optimal number of elements for this simulation as it compromised between the computational resources and the accuracy of the solution, Fig 5.

### Contact lens surfaces design

**Fig 6:**
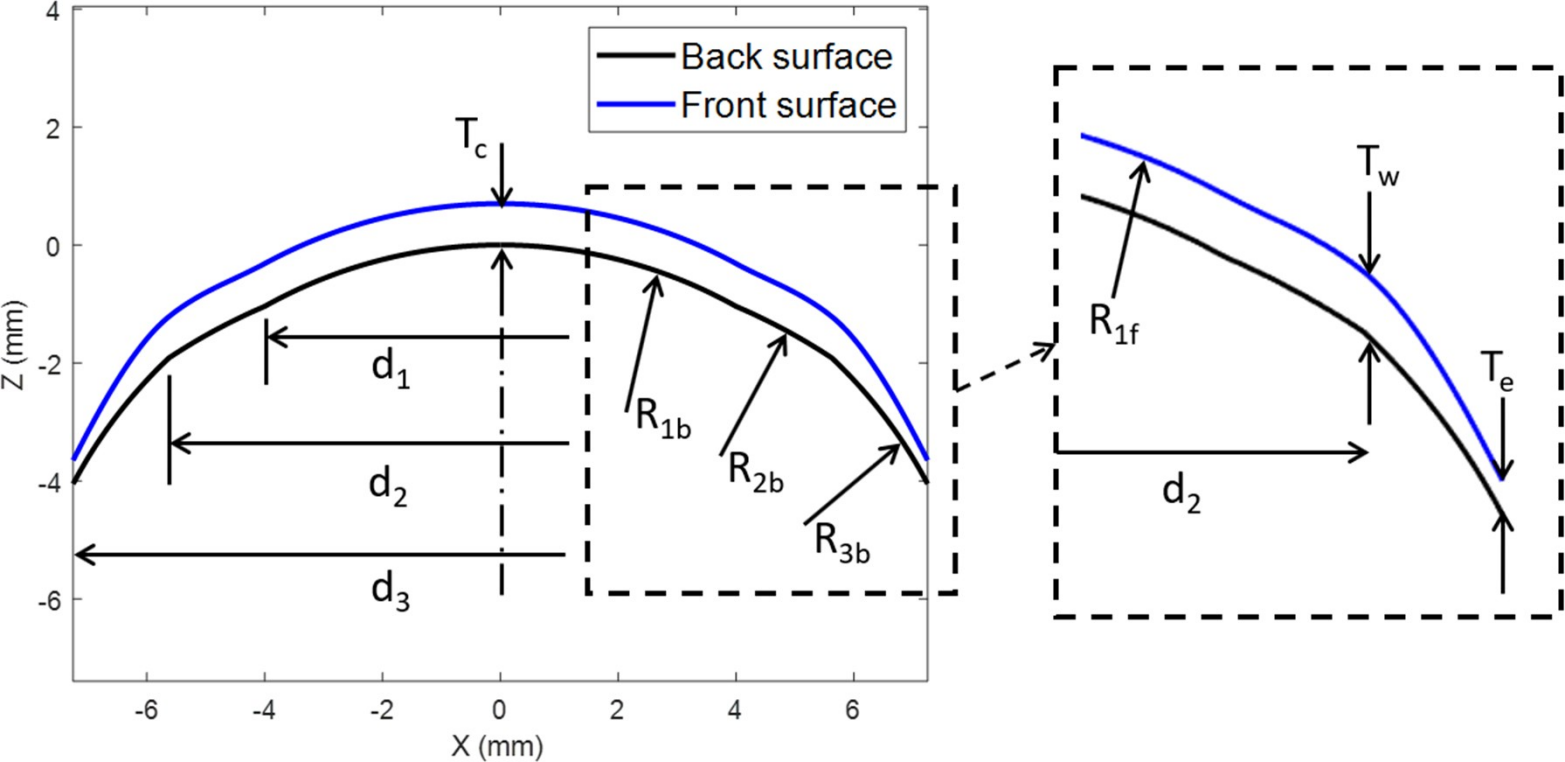
Geometry parameters of contact lenses design.

The semi-scleral contact lenses modelled in the present study were designed to mimic commercially available tri-curve lenses. The lens’ surface was divided into three zones namely, the optic zone $\left(0\leq X<\frac{d_{1}}{2}\right)$, the transient zone $\left(\frac{d_{1}}{2}\leq X<\frac{d_{2}}{2}\right)$ and the peripheral zone $\left(\frac{d_{2}}{2}\leq X\leq \frac{d_{3}}{2}\right)$, Fig 6.

A custom-made MATLAB script was prepared to generate the geometrical shape of the lenses after entering the design parameters and the optical power values. While the back-surface of the simulated contact lenses was designed to achieve a good fit with the eye topography, the front-surface was designed to achieve three goals. These goals were: 1) attain the required optical power within the optic zone; 2) add a balance ballast to the transient zone (T_w_), and 3) accomplish the required edge thickness at the end of the peripheral zone (T_e_). The optic zone is designed to cover the human eye pupil which is varying during the day depending on the level of the light to which the eye is subjected. A normal adult’s pupil size varies from 2.0 to 4.0 mm in diameter in the sunlight to 4.0 to 8.0 mm in a dark room [21], therefore, an 8.0 mm optic zone diameter ensures a working design all-over the day. The following sections describe the design of the back- and front-surfaces of each lens in detail.

### Back-surface design

Geometrical parameters considered in the design of the lens back-surface include the back optic-zone radius or the base curve (R_1b_), the back transient-zone radius (R_2b_), the peripheral curve radius (R_3b_) and the overall lens diameter (d_3_). X and Z coordinates (X_c_, Z_c_) of the centres of radii R_1b_, R_2b_ and R_3b_ were calculated as:

$$\begin{array}{c} X_{c1}=0,\;Z_{c1}=-R_{1b}\\ X_{c2}=0,\;Z_{c2}=Z_{c1}-R_{2b}\;\cos(\sin^{-1}\frac{d_{1}}{{2R}_{2b}})+R_{1b}\;\cos\left(\sin^{-1}\frac{d_{1}}{{2R}_{1b}}\right)\\ X_{c3}=0,\;Z_{c3}=Z_{c2}-R_{3b}\;\cos(\sin^{-1}\frac{d_{2}}{{2R}_{3b}})+R_{2b}\;\cos\left(\sin^{-1}\frac{d_{2}}{{2R}_{2b}}\right) \end{array}$$ Eq 4

before the back-surface height, Z_b_ was constructed as

$$Z_{b}=\left\{ \begin{array}{ccc} Z_{c1}+\sqrt{{R_{1b}}^{2}-{\left(X-X_{c1}\right)}^{2}} & , & 0\leq X<\frac{d_{1}}{2}\\ Z_{c2}+\sqrt{{R_{2b}}^{2}-{\left(X-X_{c2}\right)}^{2}} & , & \frac{d_{1}}{2}\leq X<\frac{d_{2}}{2}\\ Z_{c3}+\sqrt{{R_{3b}}^{2}-{\left(X-X_{c3}\right)}^{2}} & , & \frac{d_{2}}{2}\leq X\leq \frac{d_{3}}{2} \end{array}\right.$$ Eq 5

To reduce the number of the design parameters, the transient zone radius (R_2b_) was set to 2 mm greater than the base curve radius (R_2b_ = R_1b_ + 2). However, the peripheral zone radius R_3b_ was set to 2 mm less than the base curve radius (R_3b_ = R_1b_ – 2). The back-surface for every simulated lens was constructed meridian by meridian in three-dimensions in the anti-clockwise direction with 1° steps around the Z-axis using the MATLAB software. Unlike the front-surface, the back-surface of the simulated lenses was rotationally symmetric therefore, all back-surface meridians were identical. The range of values used in the lens geometry design is shown in Table 3, where the diameter of the balance zone ballast centre d_2_ was set to a mean value between the optic zone diameter and the overall lens diameter.

**Table 3:** Range of values used in lens design.

Base curve radius (back optic zone radius)	R_1b_	8.8, 8.5 & 8.2 mm
Optic zone diameter	d_1_	8 mm
Balance zone ballast central diameter	d_2_	11.25, 11.5 & 11.75 mm
Overall lens diameter	d_3_	14.5, 15.0 & 15.5 mm

### Front-surface design

The lens maker’s equation (Eq 6) [22] was rearranged in (Eq 7) before being used to generate the front-surface shape within the optic zone using the calculated back-surface radius (R_b_). A central lens thickness (T_c_) of 0.25 mm was used in all cases, with the exception of lenses with nominal powers of +10 D, +15 D, and +20 D. These lenses (both spherical and toric) were generated with central thicknesses T_c_ = 0.4 mm, 0.55 mm, and 0.70 mm respectively to avoid producing regions of negative volume resulting from the intersection of the front- and back-surfaces during the lens design process. The lens material refractive index (n) was set to 1.334 to simulate the hydrogel optical characteristics, however, the lens nominal power (*P*_*i*_) was varied according to the required optical power:

$$P_{i}=(n-1)\left(\frac{1}{R_{1f}}-\frac{1}{R_{1b}}+\frac{T_{c}(n-1)}{{nR}_{1f}R_{1b}}\right)$$ Eq 6

$$R_{1fi}=\frac{T_{c}{\left(n-1\right)}^{2}+n(n-1)R_{1b}}{nR_{1b}P_{i}+n(n-1)}$$ Eq 7

All front-surfaces were designed with the lens shape factor (k) set to 1.0, Therefore, the lens front-surface was shaped meridian by meridian as:

$${Z_{fi}=T}_{c}-\frac{1}{k}\left(R_{1fi}-\sqrt{{R_{1fi}}^{2}-kX^{2}}\right)$$ Eq 8

Where the subscript (*i*) stands for the meridian number and therefore *i* equals 1, 2, 3, …, 360 corresponding respectively to meridian angles θ = 0°, 1°, 359° rotating around the Z-axis in the anti-clockwise direction. The thickness of the boundary between the transient zone and the periphery zone T_w_ is calculated in a way to allow the addition of more thickness to the lower meridians (θ = 181° to 359°):

$$T_{w_{i}}=T_{c}(1-W\;\sin\theta )$$ Eq 9

where W is a weighting factor defined as:

$$W=\left\{ \begin{array}{cc} 0.2 & ,\\ 1 & , \end{array}\begin{array}{c} 0\leq \theta \leq 180\\ 180<\theta \end{array}\right.$$ Eq 10

Finally, the lens edge thickness (T_e_) was set to 0.4 mm before fitting the lens front-surface points to shape-preserving piecewise cubic interpolation [23] to ensure a smooth front-surface while keeping the designed points in their position.

### Light raytracing

**Fig 7:**
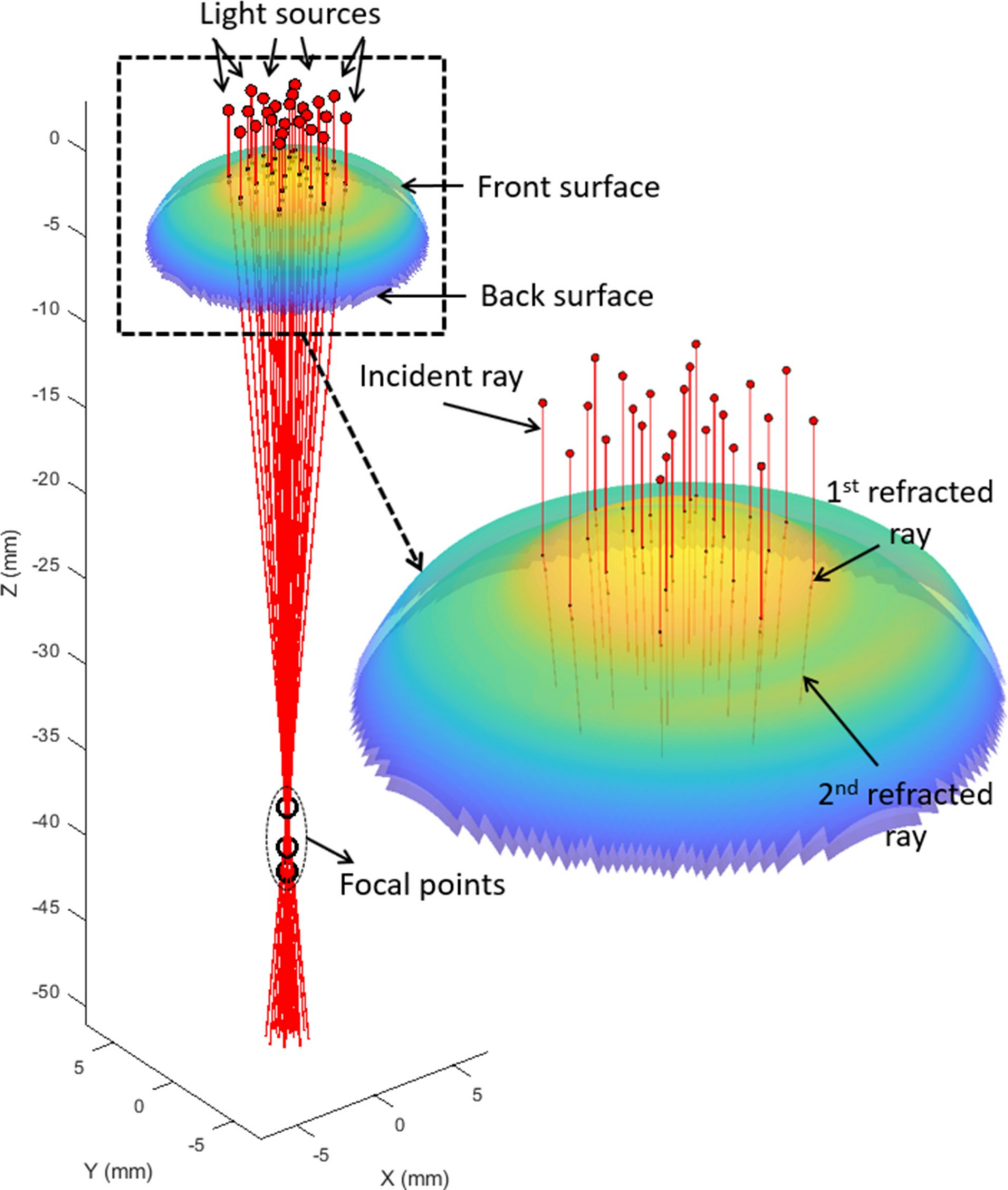
Light-ray tracing of a -20D spherical lens according to Snell's law.

To measure the EPCs that incurred by the conformance of each soft contact lens to the cornea, another technique which does not rely on the lens maker’s equation was considered. By calculating the lens power off-eye then on-eye by the light-ray tracing method, the EPC during the fitting process can be determined. Therefore, a custom-built MATLAB script performing light-ray tracing across the lens optic zone was innovated and validated using the AutoCAD software (Autodesk, Inc., San Rafael, California, USA). The light raytracing was achieved through mathematical simulation of light sources producing rays, parallel to the optical axis of the lens, directed towards the contact lens facades which were then refracted through the front- and back-surfaces following Snell's law [24]. Consequently, each light ray refracted by the lens front-surface was then used as an incident ray on to its back-surface. The focal point for each ray was then located by the point of intersection between the refracted light-ray and the lens’ optical axis. The optical power for each point on the contact lens hit by the light source rays was determined by the focal length, *f*, of each light-ray which was calculated as the distance from the lens apex to the intersection point between the second refracted light-ray and the optical axis as shown in Fig 7 [25]. In this analysis, it was noted that not all rays intersected the optical axis due to the refractive power variation in toric lenses and the phenomena of spherical aberration in spherical lenses [26]. The validated light-ray tracing script was run for each contact lens model geometry before and after the simulated fitting process to identify the EPCs and their standard deviations across the lens’ optic zone.

## Results

**Fig 8:**
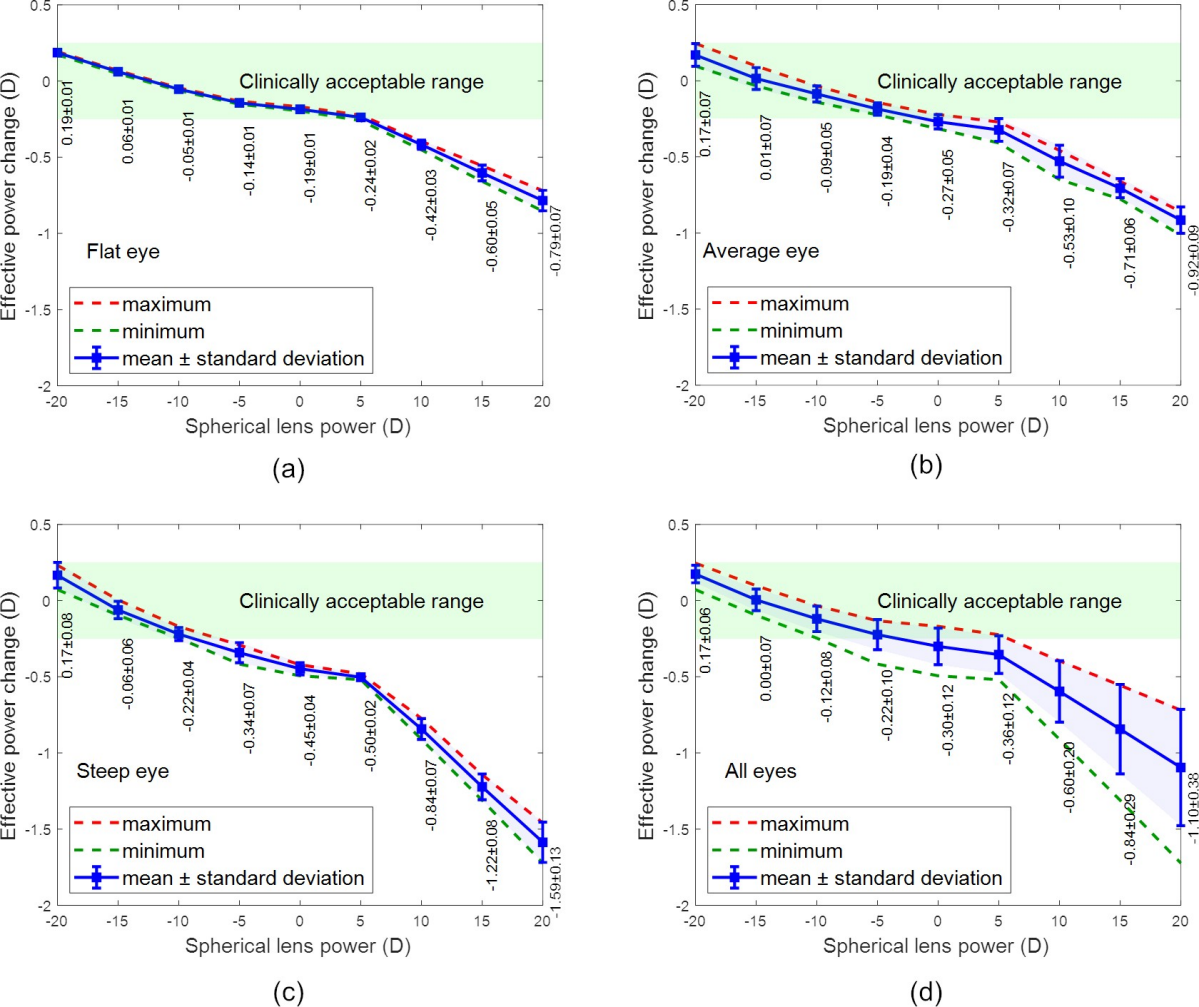
Effective power change in spherical lenses where the x-axis reports the spherical power of the contact lens when fitted to (a) a flat eye, (b) an average eye, (c) a steep eye and (d) all eyes.

**Fig 9:**
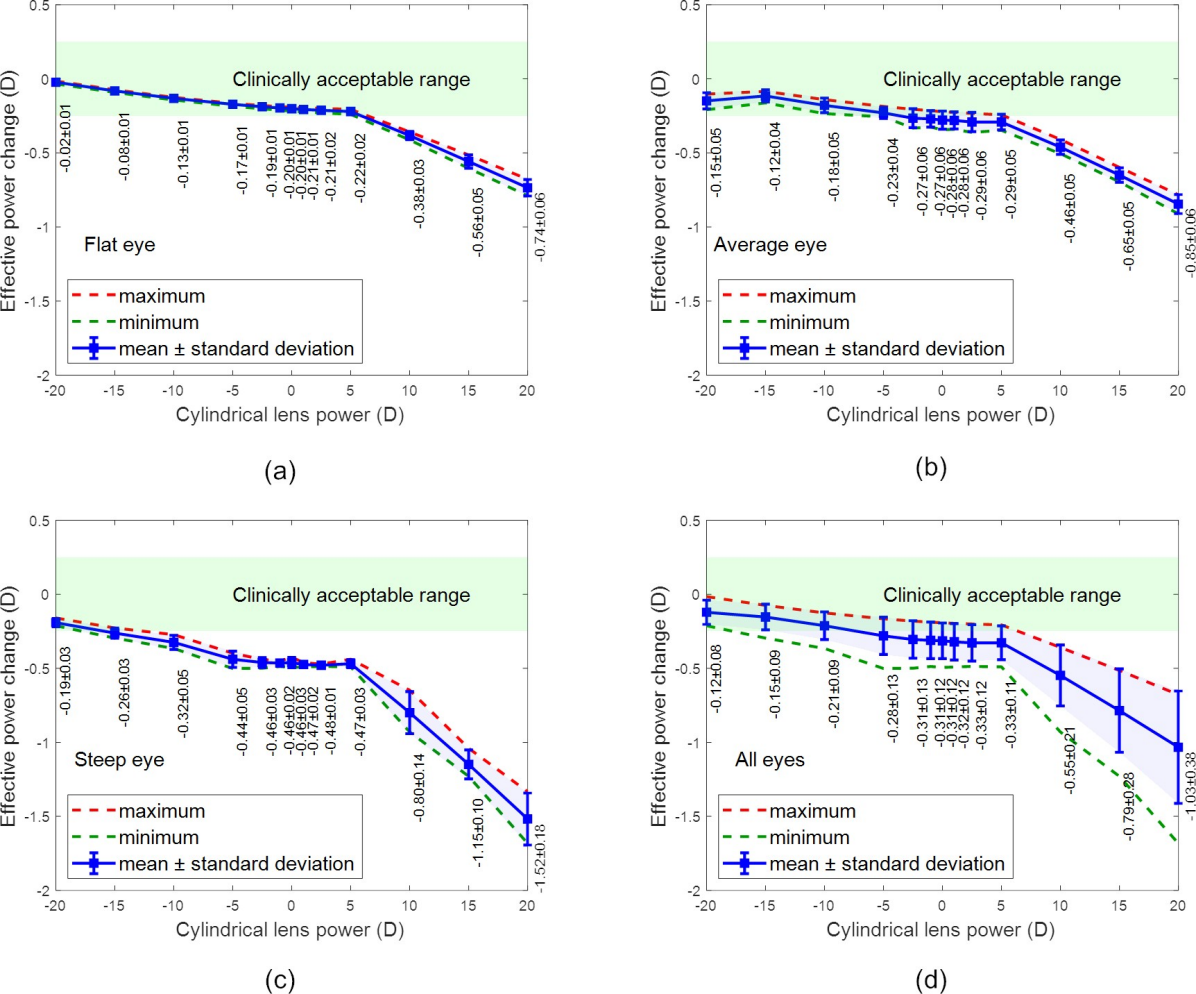
Effective power change in toric lenses where the x-axis reports the cylindrical power of the contact lens when fitted to (a) a flat eye, (b) an average eye, (c) a steep eye and (d) all eyes.

The EPCs observed for the spherical lenses are shown in Fig 8, while the toric lens results are shown in Fig 9. On average, the optical power trends show an inverse relationship between the EPC and the nominal lens power, for both spherical and toric lenses. Furthermore, in all instances, the power threshold value (i.e. the nominal lens power at which the resulting EPC becomes clinically unacceptable) displays an inverse relationship with corneal steepness. These findings are described in detail in the following subsections.

### EPC analysis

#### Spherical lenses

Fig 8A to 8C contains the results for spherical lens simulations on flat, average and steep corneas. In measuring the EPCs for each simulation, the values observed over the nominal lens power range of -20 to +20 D was found to reduce from 0.19 ± 0.01 to -0.79 ± 0.07 D, 0.17 ± 0.07 to -0.92 ± 0.09 D and 0.17 ± 0.08 to -1.59 ± 0.13 D for the flat, average and steep corneas, respectively. For nominal lens designs between -20 and +5 D, the value of the EPC reduced non-linearly with the slope reducing as the nominal power increased. However, from +5 to +20 D, the change becomes linear and the rate of change increased. Additionally, as the steepness of the cornea increased, the power threshold value was found to reduce from 5 D for a flat cornea to 0 D for an average cornea and finally to -8 D for a steep cornea. The average values of all spherical lens simulations are presented in Fig 8D.

The results show that the EPCs in spherical lenses are less important in negative power lenses, however, the power threshold value is dependent on the eye’s shape. EPCs of a spherical lens on a flat eye does not need to be corrected unless the nominal lens power is greater than +5 D. However, a spherical lens for a steep eye may need to be corrected if its nominal power is greater than -5 D.

#### Toric lenses

Similar to the spherical lenses, from which it can be observed that EPCs are most significant near the extremes of the prescription spectrum for spherical lenses. However, there appear to be fewer clinically significant EPCs in the negative prescriptions for toric lenses than the negative prescriptions for spherical lenses. For the toric lenses, the EPCs observed between CYL = -15 D and CYL = +5 D are largely negligible.

As addressed above, it is thought that the additional central thickness required in the high-power positive lenses CYL = +10 D, +15 D, and +20 D (Tc = 0.40 mm, 0.55 mm, and 0.70 mm respectively) is likely to be the reason the positive EPCs are noticeably greater than those on the negative end of the spectrum.

In measuring the maximum and minimum power changes for each toric lens simulation, values ranged from a maximum of +1.501 ± 0.338 D (Average Eye, SPH = -20 D, R_1b_ = 8.5 mm, d_3_ = 15.0 mm), to a minimum of -3.514 ± 0.731 D (Steep Eye, SPH = +20 D, R_1b_ = 8.2 mm, d_3_ = 14.5 mm).

Similar to the spherical lenses, the toric contact lenses of high positive and negative prescriptions demonstrated larger effective, minimum, and maximum power changes on fitting than the lower prescriptions. Again, this is likely to be attributable to the extreme geometries of these lenses being more affected by conformance to the cornea.

Contrary to the spherical lenses, the EPCs observed between SPH = -15 D and SPH = +5 D are clinically negligible for toric lenses. Spherical lenses appear therefore to be more affected by optical power changes during fitting than toric lenses for simple astigmatism.

### Parametric analysis

#### Spherical lenses

**Fig 10:**
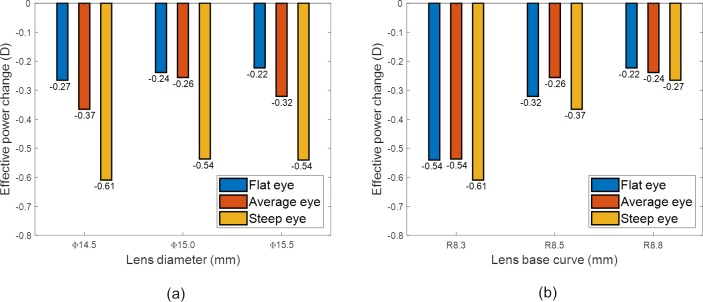
Parametric analysis of spherical prescriptions, (a) effect of lens diameter, (b) effect of the lens base curve.

The results of Fig 8 were analysed parametrically, to assess the impact of the lens base curve (R_1b_) and diameter (d_3_) on EPC. Simulated lenses for fitting flat, average and steep eyes were categorised according to their diameter (d_3_) and plotted against the EPC in Fig 10A where the result shows that the lenses diameter (d_3_) has a very limited effect on the EPC among the flat, average and steep eyes. As the observed difference in the EPC was always less than 0.25 D, there is no clinical need to correct the lens power in the prescription according to the lens diameter (d_3_).

When simulated lenses for fitting flat, average and steep eyes were categorised according to their base curves (R_1b_) in Fig 10B, the results showed generally that the bigger the base curve the less EPC, however, the average eye only recoded EPCs bigger than 0.25 D between R_1b_ = 8.3 mm and R_1b_ = 8.5 mm. No further clinically correctable EPCs were observed when the base curve was increased from R_1b_ = 8.5 mm to 8.8 mm.

#### Toric lenses

**Fig 11:**
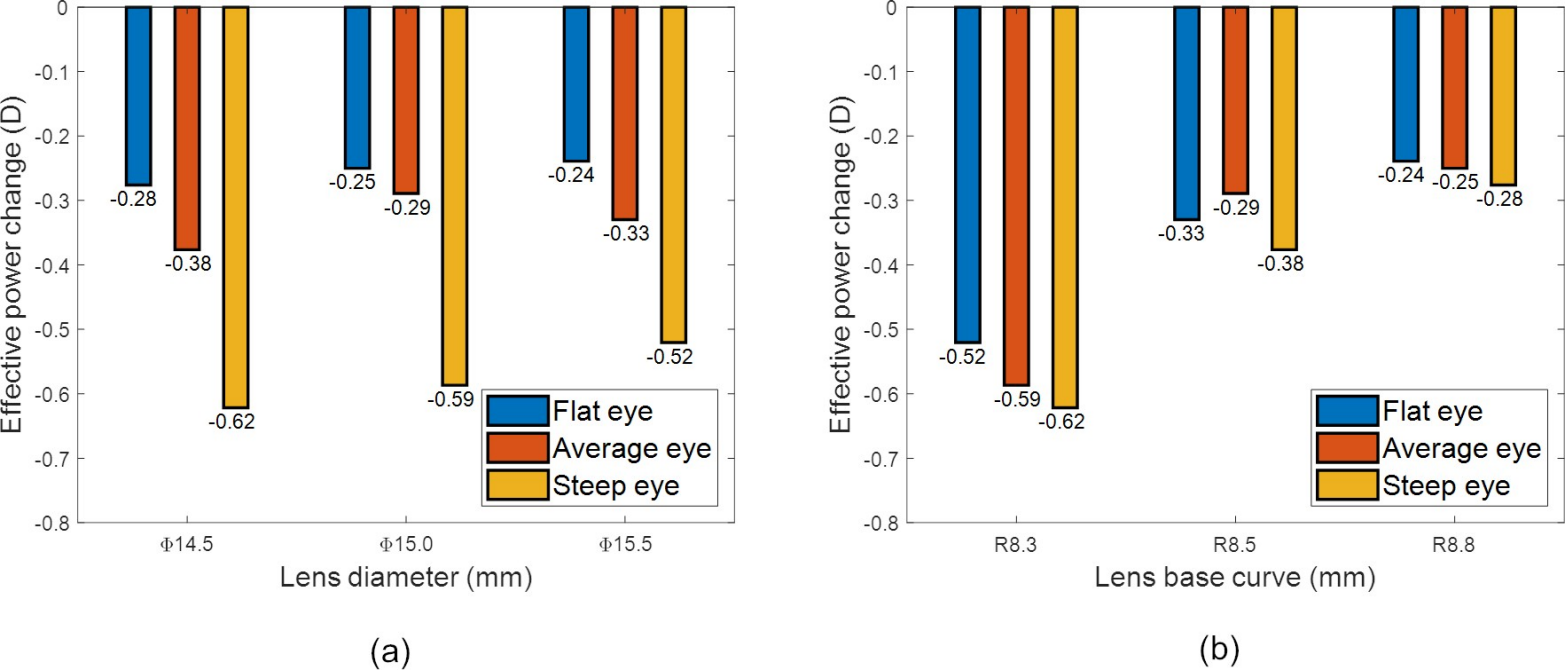
Parametric analysis of toric prescriptions, (a) effect of lens diameter, (b) effect of the lens base curve.

The results of Fig 9 were analysed, to assess the impact of the lens base curve and diameter on EPC for the toric lenses, Fig 11. The observed impacts were similar to those of the spherical lenses where there were no clinically correctable EPCs observed when the lens diameter was increased from 14.5 mm to 15.5 mm, however only the average eye showed correctable EPCs bigger than 0.25 D when the base curve was changed from R_1b_ = 8.3 mm to R_1b_ = 8.5 mm. The impact of the base curve on EPC was consistent across the entire range of nominal toric prescriptions, the steepest base curves yielding the largest magnitude EPC, and the flattest base curves were the smallest.

## Discussion

Unlike spectacle lenses, which are used in conventional glasses, soft contact lenses should be designed to fit the user’s eyes besides being able to correct his/her vision. With the lack of a clear design role in the soft contact lens industry, the companies working in this field have developed empirical methods to design and fit soft contact lenses. In this study, a clear design equation based on mathematical and geometrical analyses was provided and tested via computer simulation. When a contact lens is placed on the eye, the effects of the eyelid interaction and the tears’ surface tension change the lens dimensions and therefore alters its predesigned refractive power. Power changes occur as the surface of a lens undergoes relative changes in shape due to interaction with the cornea. With this interaction the optical path through the lens is changed, consequently changing the focal length and power throughout the lens’ optic zone. This study considered the impact of soft contact lens design parameters on the EPC after eye-fit. Since the clinical visual acuity test is carried out at a distance of 6.0 m which induces a 0.167 D accommodation, this power is considered as clinically negligible. Therefore, the trial lenses are in 0.25 D steps and EPCs were considered to be clinically acceptable if |ΔP| ≤ 0.25 D [27, 28].

Soft lenses of steeper base curves and smaller diameters showed generally to be more susceptible to clinically significant (> 0.25 D) EPCs, particularly for strong positive (> +5 D) nominal powers. Both spherical and toric (simple astigmatism) lenses demonstrated similar trends in EPCs on fitting from -20 D to +20 D, with EPCs increasing negatively as nominal lens power increases positively.

The positive lenses simulated on the high end of the spectrum, SPH = +10 D, +15 D, and +20 D, had to be designed thicker than the rest, with Tc = 0.40 mm, 0.55 mm, and 0.70 mm, respectively. The additional thickness was necessary to ensure no negative volumes were generated in the lens cross-section as a result of the high positive power requirements of these lens designs. Thicker lenses for +10 D, +15 D, +20 D likely affected the slope of EPC curves and results for these measures, but the general trend still stands.

It follows that spherical contact lenses of high positive and negative prescriptions demonstrate larger effective, minimum, and maximum power changes on fitting than the more minor prescriptions. This is likely attributable to the fact that lenses of a higher power (both positive and negative) are thicker and require a more extreme front-surface design through which to refract light and shorten the optical path. Consequently, their more extreme geometries may be more extremely affected by conformance to the cornea. This higher amount of power changes in the higher degrees are particularly relevant in clinical practice. Fitting contact lenses in these patients is usually more challenging, time-consuming and includes higher costs to replace the lens with possible incorrect degrees due to the change in its power [29]. Since there is a wide range of contact lenses available off-the-shelf at local opticians and online, and most of the patients do not have a regular follow-up visit to check their contact lenses fit, problems related to the incorrect effective power of the contact lens, like asthenopia, may go unnoticed [30, 31]. The wearing of glasses specifically in high myopes can limit their ability to pursue outside activities and as consequence lead to worsening of myopia [32, 33].

A more accurate estimation of the EPC would also help the contact lenses fitting for children with a high degree of myopia or hyperopia, who often cannot provide useful verbal information for a good over-refraction exam [34, 35].

Some limitations of the study are the design of a conceptual study with the inclusion of three eyes, the use of one type of lens design and material. Different materials on the market, like the silicone hydrogel, are relatively more stiff than the hydrogel used in this study, therefore the results would not be directly applied to them, however, they are expected to give the same trends with a slight shift in EPC values. Contact lens with stiffer materials could represent a better option for fitting in high ametropias [9]. Future perspectives to implement the results of this study in clinical practice are to model different specific commercially available lens designs along with a sample of contact lens wearers considered in addition to the normal corneal curvature distribution, normal variations of corneal asphericity, diameter and refractive errors. This will allow to a patient-specific recommendation of the lens shape and power prescription, according to the options available on the market.

## Supporting information

S1 Table Eyes’ clinical data. (XLSX)

S2 Table Spherical lenses’ data. (XLSX)

S3 Table Toric lenses’ data. (XLSX)

## References

[1] Holden BA, Siddle J, Robson G, Zantos S. Soft Lens Performance Models: The Clinical Significance of the Lens Flexure Effect. The Australian Journal of Optometry. 1976;59:117–29.

[2] Sarver M, Harris M, Polse KA. Corneal curvature and supplemental power effect of the Bausch and Lomb SOFLENS contact lens. Am J Optom Physiol Opt. 1975;52(7):470–3. 1180322 10.1097/00006324-197507000-00004

[3] Plainis S, Charman WN. On-Eye Power Characteristics of Soft Contact Lenses. Optometry and Vision Science. 1998;75(1):44–54. 9460786 10.1097/00006324-199801000-00024

[4] Dietze HH, Cox MJ. On- and Off-Eye Spherical Aberration of Soft Contact Lenses and Consequent Changes of Effective Lens Power. Optometry and Vision Science. 2003;80(2):126–34. 12597327 10.1097/00006324-200302000-00008

[5] Bennett AG. Optics of Contact Lenses 5 ed London, UK: Association of Dispensing Contacts; 1985. 99 p.

[6] Weissman BA, Gardner KM. Power and Radius Changes Induced in Soft Contact Lens Systems by Flexure. American Journal of Optometry & Physiological Optics. 1984;61(4):239–45. 6547274 10.1097/00006324-198404000-00003

[7] Strachan JPF. Some Principles of the Optics of Hydrophlllc Lenses and Geometrical Optics Applied to Flexible Lenses. The Australian Journal of Optometry. 1973;56(1):25–33. 10.1111/j.1444-0938.1973.tb01065.x

[8] Whittle J. Contact lens optics and lens design. Eye. 2007;21:1022 10.1038/sj.eye.6702607

[9] Kollbaum PS. Comparing the Optical Properties of Soft Contact Lenses On and Off the Eye. Optom Vis Sci. 2013;90(9):924–36. 10.1097/01.opx.0000434275.93435.da 23969894 PMC3902057

[10] Sulley A, Lorenz KO, Wolffsohn JS, Young G. Theoretical fitting characteristics of typical soft contact lens designs. Contact Lens & Anterior Eye. 2017;40:248–52. 28501442 10.1016/j.clae.2017.04.001

[11] Young G. 8—Soft Lens Design and Fitting. Efron N, editor: Elsevier; 2018.

[12] Gilani F, Cortese M, Ambrosio R Jr, Lopes B, Ramos I, Harvey EM, et al Comprehensive anterior segment normal values generated by rotating Scheimpflug tomography. Journal of cataract and refractive surgery. 2013;39:1707–12. 10.1016/j.jcrs.2013.05.042 24054966

[13] Gasson A, Morris JA. The Contact Lens Manual: A Practical Guide to Fitting. 4 ed Great Britain Elsevier Health 2010.

[14] Davis JR. Tensile Testing. 2 ed: ASM International; 2004.

[15] Shaw AJ, Collins MJ, Davis BA, Carney LG. Eyelid pressure and contact with the ocular surface. Investigative ophthalmology & visual science. 2010;51(4):1911–7. 10.1167/iovs.09-4090 . 19834035

[16] Zhao G, Wollmer P. Surface activity of tear fluid. Acta Ophthalmol Scand. 1998;76:438–41. 9716330 10.1034/j.1600-0420.1998.760409.x

[17] Kalogeropoulos G, Chang S, Bolton T, Jalbert I. The effects of short-term lens wear and eye rubbing on the corneal epithelium. Eye Contact Lens. 2009;35(5):255–9. Epub 2009/08/07. 10.1097/ICL.0b013e3181b4ec39 . 19657277

[18] Yap TE, Archer TJ, Gobbe M, Reinstein DZ. Comparison of Central Corneal Thickness Between Fourier-Domain OCT, Very High-Frequency Digital Ultrasound, and Scheimpflug Imaging Systems. J Refract Surg. 2016;32(2):110–6. Epub 2016/02/10. 10.3928/1081597X-20151223-01 . 26856428

[19] Sterner O, Aeschlimann R, Zürcher S, Osborn Lorenz K, Kakkassery J, Spencer ND, et al Friction Measurements on Contact Lenses in a Physiologically Relevant Environment: Effect of Testing Conditions on Friction. Investigative ophthalmology & visual science. 2016;57(13):5383–92. 10.1167/iovs.16-19713 27737459

[20] Day KD. The Mechanics of a Hydrogel Contact Lens on the Human Eye with a Lubricating Tear Layer. USA: Massachusetts Institute of Technology; 1997.

[21] Walker HK, Hall WD, Hurst JW. Clinical Methods: The History, Physical, and Laboratory Examinations. Portland: Butterworths; 1990. 21250045

[22] Wolfe WL. Introduction to Imaging Spectrometers. USA: SPIE; 1997.

[23] Yang L, Huiyan Z. Shape preserving piecewise cubic interpolation. Applied Mathematics. 1996;11(4):419–24. 10.1007/BF02662881

[24] Khurana AK. Theory And Practice Of Optics And Refraction. 3 ed India: Elsevier India Pvt. Limited; 2008.

[25] Wang L, Mahmoud AM, Anderson BL, Koch DD, Roberts CJ. Total corneal power estimation: ray tracing method versus gaussian optics formula. Investigative ophthalmology & visual science. 2011;52(3):1716–22. 10.1167/iovs.09-4982 . 21071742

[26] Welford WT. Aberrations of optical systems: CRC Press, Taylor & Francis; 1986.

[27] Schiefer U, Kraus C, Baumbach P, Ungewiß J, Michels R. Refractive errors: Epidemiology, Effects and Treatment Options. Deutsches Ärzteblatt International. 2016;113(41):693–702. 10.3238/arztebl.2016.0693 PMC5143802. 27839543 PMC5143802

[28] Cunha CC, Berezovsky A, Furtado JM, Ferraz NN, Fernandes AG, Muñoz S, et al Presbyopia and Ocular Conditions Causing Near Vision Impairment in Older Adults From the Brazilian Amazon Region. American Journal of Ophthalmology. 2018;196:72–81. 10.1016/j.ajo.2018.08.012 30118685

[29] Astin CL. Contact lens fitting in high degree myopia. Cont Lens Anterior Eye. 1999;22 Suppl 1:S14–9. Epub 2005/11/24. . 16303419 10.1016/s1367-0484(99)80038-1

[30] Ky W, Scherick K, Stenson S. Clinical survey of lens care in contact lens patients. CLAO J. 1998;24(4):216–9. Epub 1998/11/04. . 9800060

[31] Chalmers RL, Keay L, Long B, Bergenske P, Giles T, Bullimore MA. Risk factors for contact lens complications in US clinical practices. Optom Vis Sci. 2010;87(10):725–35. Epub 2010/08/24. 10.1097/OPX.0b013e3181f31f68 . 20729772

[32] Recko M, Stahl ED. Childhood myopia: epidemiology, risk factors, and prevention. Mo Med. 2015;112(2):116–21. Epub 2015/05/12. 25958656 PMC6170055

[33] Cooper J, Tkatchenko AV. A Review of Current Concepts of the Etiology and Treatment of Myopia. Eye Contact Lens. 2018;44(4):231–47. Epub 2018/06/15. 10.1097/ICL.0000000000000499 29901472 PMC6023584

[34] Walline JJ, Long S, Zadnik K. Daily disposable contact lens wear in myopic children. Optom Vis Sci. 2004;81(4):255–9. Epub 2004/04/21. . 15097767 10.1097/00006324-200404000-00011

[35] Schulle KL, Berntsen DA, Sinnott LT, Bickle KM, Gostovic AT, Pierce GE, et al Visual Acuity and Over-refraction in Myopic Children Fitted with Soft Multifocal Contact Lenses. Optom Vis Sci. 2018;95(4):292–8. Epub 2018/03/22. 10.1097/OPX.0000000000001207 29561497 PMC5880703

